# The need for genetically‐informative designs in developmental science: Commentary on Gustavson et al. (2021) and Myers et al. (2021)

**DOI:** 10.1002/jcv2.12025

**Published:** 2021-08-04

**Authors:** Mark J. Taylor

**Affiliations:** ^1^ Department of Medical Epidemiology and Biostatistics Karolinska Institutet Stockholm Sweden

**Keywords:** ADHD, autism spectrum disorders, siblings, twins

The papers in the July issue of *JCPP Advances* from Gustavson et al. and Myers et al. draw further attention to the critical issue of familial confounding. Familial confounding has been known to be an important consideration in epidemiology for some time now. Many supposedly environmental risk factors are themselves heritable, the so‐called ‘nature of nurture’ (Plomin & Bergeman, 1991). Some of the genetic and shared environmental risks that influence a particular outcome may themselves also be causes of certain exposures shown to be associated with that outcome, thus confounding their association. While randomized clinical trials provide a gold standard for addressing confounding and establishing causal effect, such study designs are unfeasible or unethical for most potential risk factors. As such, powerful alternative study designs are needed, such as studies that utilize differentially exposed relatives (or include individuals discordant for the outcome), to test whether an association persists beyond familial confounding.

The issue is nicely illustrated in Gustavson et al.'s article. They note that prior studies have reported an association between maternal use of acetaminophen (i.e., paracetamol) during pregnancy and attention‐deficit/hyperactivity disorder (ADHD) in their children. However, both of these phenotypes are known to be heritable, and so they tested whether their association was due to familial confounding. They used data from the population‐based Norwegian Mother, Father, and Child Birth Cohort Study (MoBa), which recruited pregnant women in Norway between 1999 and 2008. Mothers were asked to report on medications they had used for medical conditions at gestational weeks 17 and 30, and again 6 months after giving birth. ADHD diagnoses were ascertained from the Norwegian Patient Register.

Their results provide a striking illustration of familial confounding. They found that although short‐term use of acetaminophen during pregnancy was not associated with an increased risk for ADHD, longer term use (defined as over 29 days) was associated with an approximately twofold increase in the risk of ADHD. On face value, then, these results could be taken to mean that using acetaminophen during pregnancy could increase the risk of ADHD in children. However, they then studied this association within sibling pairs where mothers had taken acetaminophen during one pregnancy but not the other. The association attenuated drastically in these analyses, thus suggesting that familial confounders are likely to explain the association between maternal acetaminophen during pregnancy and ADHD in children. As acetaminophen was most often reported to be used to manage pain, it is possible that these results reflect shared causes between pain disorders and ADHD.

Gustavson et al.'s findings are important, particularly for helping clinicians to provide accurate information to pregnant women about the risks associated with particular medications during pregnancy. Similar studies have been conducted for other medications during pregnancy, such as SSRIs, and report supportive results (Morales et al., 2018). Gustavson et al.'s results do, however, highlight an important cautionary note when it comes to conducting sibling comparisons. Their initial sample size was very large. However, in a sibling comparison design, only the sibling pairs who are discordant for exposure and outcome contribute to the analyses. As a consequence, and as seen in Gustavson's paper, the sample size soon declines. Accordingly, confidence intervals are very wide for the sibling analyses, and so conclusions around familial confounding should be drawn with this in mind. Studies that pool data from multiple cohorts and perform meta‐analyses of the results across cohorts have the potential to provide solutions to any issues with statistical power that may arise (see e.g., Huybrechts et al., 2018).

Siblings are not the only relatives that can be useful in genetically informative designs. Myers et al. draw attention to the within twin‐pair design. The value of twin studies in disentangling genetic and environmental influences on a trait has been known for some time now. While twin studies are often used to estimate the heritability of a given trait, and the genetic correlations between multiple traits, monozygotic (MZ) twins can provide insights into potential causal links between two phenotypes. They share almost all of their segregating DNA code, as well as some environmental exposures. As a result, any within‐pair difference between MZ twins is potentially due to environmental factors that contribute to differences between the twins or may reflect causal effects. Myers et al. highlight the discordant MZ twin design in particular, in which the association between MZ differences on two traits can be tested. They conducted an impressive systematic review of such studies in relation to autism. They note that while autism is under strong genetic influence, twin studies support a modest contribution from non shared environment.

Their review ultimately included 26 studies, which focused on the association between MZ differences in autism, autistic symptoms, and autistic traits, and four broadly defined groups of measures: genetics, brain, cognition, and physical problems. Many of the studies that they included utilized data collected from participants in the Roots of Autism Twin Study in Sweden (RATSS), one of the largest samples of twins selected due to their discordance for autism or autistic traits. They note that studies focusing on discordant twins (or examining within twin‐pair differences) report different DNA methylation in MZ twins discordant for autism, an association with neural connectivity and volume, social cognition, and physical symptoms. There are, notably, some inconsistencies in the results, however, indicating that certain results are currently not replicating. Of note, available sample sizes are small; Myers et al. include some case studies in their review, for example. It is thus clear that larger studies, such as RATSS, will be needed in future studies of discordant twin pairs in order to robustly investigate within twin pair differences.

Overall, the studies reviewed by Myers et al. highlight that this particular design may be a useful tool for better understanding the non‐shared environmental basis of autism, and they provide some very useful recommendations for analyzing discordant twin pair data and for reporting such studies. This optimism, however, should be tempered with the limitation that certain exposures cannot be meaningfully studied within twin pairs, since some are completely shared between the twins. As one example, advanced paternal age has been shown to be associated with neurodevelopmental and psychiatric disorders (D'Onofrio et al., 2014). While sibling comparisons indicate that such as an association persists after adjustment for familial confounding (D'Onofrio et al., 2014), such a test could not be conducted in twins. As such, there is a need for multiple methodological approaches, such as sibling designs and instrumental variable designs, to triangulate findings from study designs with different strength and limitations. Within twin‐pair designs are likely to have even greater issues with statistical power than sibling comparisons, as twins are somewhat rare and, as for siblings, only pairs discordant for exposure and outcome contribute to a given analysis.

The work by Gustavson et al. and Myers et al. shows just how important it is to consider familial confounding in epidemiological research. However, Myers et al. also review studies that include measurements from neuroscience and developmental science, thus highlighting the need to consider familial confounding in multiple fields of research. The designs they utilize or review are clearly important from a public health standpoint in disentangling the potential shared etiological basis of particular pairs of exposures and outcomes. A reported association between maternal acetaminophen use during pregnancy and offspring ADHD has the potential to alarm expectant mothers, for example; assessing whether such associations are due to familial confounding can help to provide more accurate information to members of the public about the risks associated with particular exposures. Readers should take heed, however, of the need for large samples in such research and of the fact that even large population‐based studies can sometimes identify few informative sibling pairs for analysis. In order to help with the interpretation of such studies, researchers using this design would do well to consider reporting the number of informative sibling pairs, as was the case in Gustavson et al.'s paper and as recommended by Myers et al.

It is also notable that both articles focus on autism and ADHD. A large quantity of epidemiological research focuses on these conditions, despite the fact that neurodevelopmental disorders constitute a far broader group of disorders, including (but not limited to) intellectual disability, dyspraxia, and dyslexia. A systematic review of sibling‐based designs published in 2020 attempted to review genetically informative studies of neurodevelopmental disorders other than autism and ADHD concluded that very few studies had focused on such conditions, meaning that knowledge about the etiology of these disorders is lacking in comparison to that about autism and ADHD (Carlsson et al., 2020). Many of these disorders are not necessarily rarer than autism or ADHD either. A UK population‐based study reported that the prevalence of language disorders with no known origin was 7.58%, for example, which is substantially higher than the reported prevalence for autism and similar to prevalence rates reported for ADHD (Norbury et al., 2016). Given that other neurodevelopmental disorders appear to have similar heritability estimates to autism and ADHD, the designs highlighted by Gustavson et al. and Myers et al. have great potential to cast light on the causes of disorders about which little is known.

## CONFLICT OF INTEREST

The author is a member of the Editorial Advisory Board for JCPP *Advances*. [Corrections made on 22 June 2022, after first online publication: This Conflict of Interest statement has been corrected in this version.]

## Data Availability

The data that support the findings of this study are available from the corresponding author upon reasonable request.
